# Editorial: Case reports in hypertension: 2022

**DOI:** 10.3389/fcvm.2023.1210740

**Published:** 2023-05-22

**Authors:** Guido Iaccarino

**Affiliations:** Center for Research on Hypertension and Related Conditions, Federico II University of Naples, Naples, Italy

**Keywords:** case report, secondary hypertension, malignant hypertension (MHT), hyperaldosteronism, nefrovascular hypertension

**Editorial on the Research Topic** Case reports in hypertension: 2022

## 1. Introduction

Clinical guidelines for the management of hypertension are regularly updated, and the whole community of hypertension specialists is longing for the promised new guidelines of the ESH/ESC expected to be released in June 2023. Despite the availability of such important reference points, in daily practice, the management of hypertension often requires refined clinical skills. This is particularly true when facing peculiar hypertensive cases such as resistant hypertension or hypertension of the young, particularly in non-obese patients. In these cases, the possibility to diagnose a secondary form of hypertension is particularly high but requires that all the possible causes are explored, even those that are less frequent. This is when the investigative capabilities of the clinician come to play only if supported by a profound knowledge of the physiopathology of the disease.

**Figure 1:**
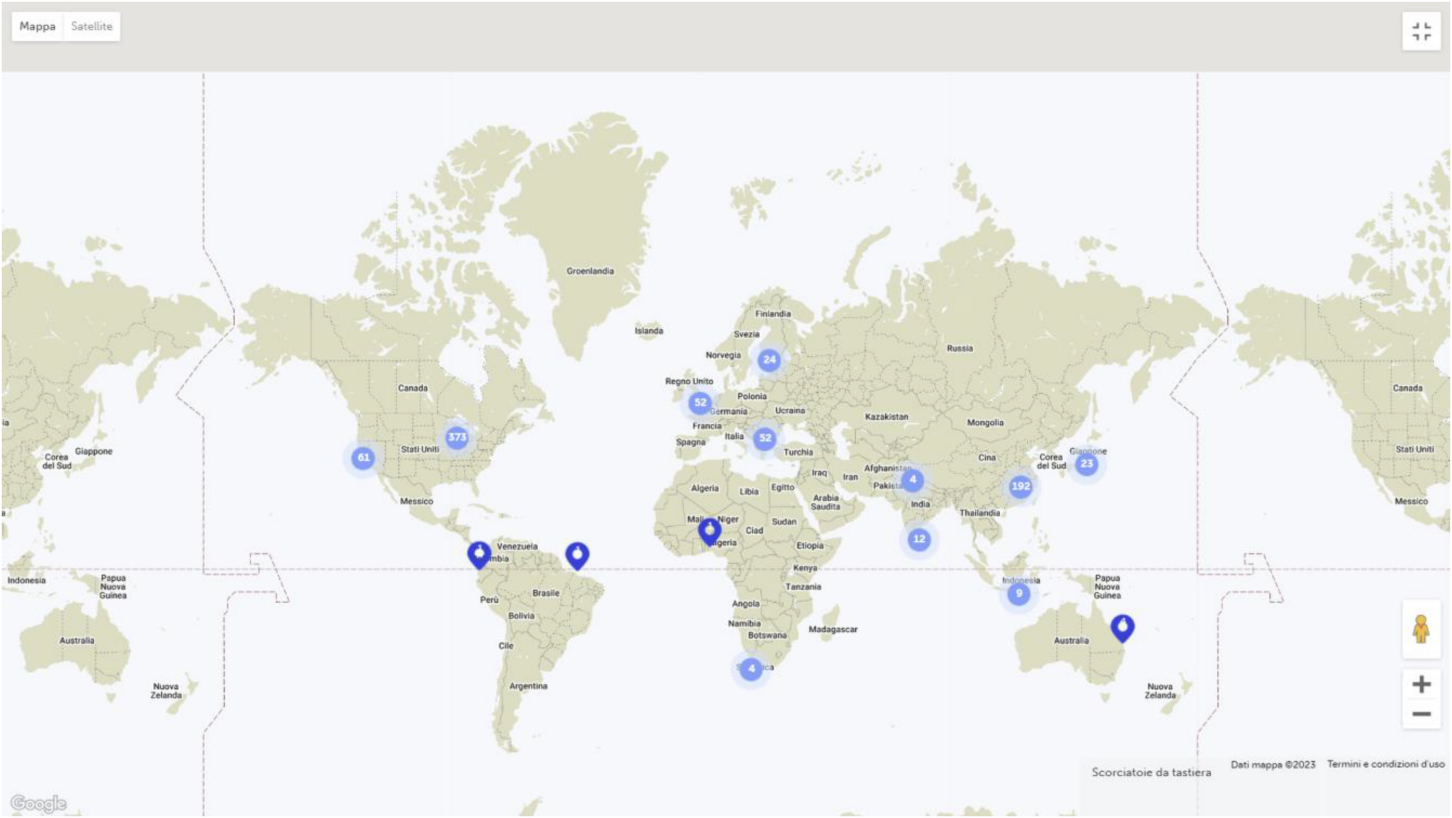
Impact of the research topic: the number of downloads of papers on this research topic from around the world since 2022.

In this series of clinical cases in hypertension, five papers were published from January 2022 to January 2023. Again, the relevance of a similar Research Topic is confirmed by its impact around the world (Figure 1). The treated cases focus on specific cases, which are sometimes neglected by the guidelines for the lack of specific clinical trials. Two cases approach the adrenal gland as causative of hypertension, one discusses the renal artery, and two more identify an oncological cause of the disease with very well-hidden tumors. Altogether, these cases point to emerging clinical conditions in hypertension and the possibility of novel technology for their management.

## 2. A modern approach to classifying renal artery fibrodysplasia

Fibromuscular dysplasia (FMD) is the second most common etiology of renal artery stenosis (RAS) and renovascular hypertension, especially in young patients (1). FMD is a systemic arterial disease presenting as arterial stenosis, aneurysm, and dissection in virtually any arterial bed. When affecting the renal artery, FMD causes significant cardiovascular manifestations including renovascular hypertension. Although several studies have demonstrated the benefits of renal artery revascularization, it is difficult to predict the real benefit of this procedure, and there is evidence that appropriate patient selection is, therefore, the key to avoiding therapeutic failure (2). In this report, Yuxi Li et al. borrow a hemodynamic assessment technology from coronary intervention using the combination of pressure guidewire [to calculate Flow Fractional Reserve (FFR)] and coherence tomography (OCT)-guided angioplasty treatment in a young hypertensive patient with renal FMD Li et al. The authors walk us through the interpretation of the data received from the combination of both technologies. In particular, OCT helps in confirming the diagnosis of the angiographically ambiguous lesion, properly classifying FMD, and guiding and optimizing the intervention. The FFR before and after the procedure demonstrated the efficient therapy of the stenosis with a significant reduction and normalization of the pressure gradient. The combined use of OCT and rFFR may improve the diagnosis and stratification of patients, and it will help identify patients who may respond to revascularization and guide the procedure.

## 3. Superselective adrenal arterial embolization in primary aldosteronism

Approximately 4%–5% of patients undergoing abdominal imaging report adrenal incidentaloma, with an increase in the number of patients in the age group. A similar percentage (6%) is reported in a series of 87,065 autopsies (3). Interventional radiology of the adrenal glands represents an important interventional radiologic procedure indicated during primary aldosteronism (PA). Hokotate et al. (4) reported that 33 cases of aldosteronism were treated with embolization. The results were encouraging. The plasma levels of aldosterone returned to normal in 82% of patients. In this issue, the case report of a successful selective embolization of the adrenal adenoma in a 39-year-old man with a 5-year history of hypertension was discussed Zhou et al.

## 4. Primary Aldosteronism and Subclinical Cushing Syndrome

Over the years, the approach to identifying, diagnosing, and treating primary aldosteronism has remained unchanged and often focuses on detecting and treating the more severe symptoms of the condition. However, recent evidence shows that the prevalence of primary aldosteronism is higher than previously thought and that the small and abnormal production of aldosterone without renin alteration may cause an increase in cardiovascular risk (5). The CoSh syndrome (synchronous adrenal aldosterone and excess cortisol) is a distinct entity in PA, as described by Arlt et al. in 2017 (6). The disease has been studied for more than 40 years. Recently, this has become one of the most dynamic topics in hormonally active adrenal lesions due to significant progress in steroid metabolomics, molecular genetics of the immune CYP11B1/B2 gene constellation, and newly developed pathological types in the 2022 WHO classification (6). Lihua Hu et al. described the management of a 49-year-old woman with coexisting primary aldosteronism (PA) and subclinical Cushing's syndrome due to a right adrenal adenoma. Hu et al.

## 5. Pheochromocytoma of the urinary bladder

According to existing literature, pheochromocytomas account for 0.05%–0.1% of patients with hypertension (7). Although paroxysmal or resistant hypertension is still a major cause for the diagnostic workup of pheochromocytoma, there is a steadily increasing proportion of cases found as part of investigations for adrenal incidentalomas or accidentally found extra-adrenal paragangliomas showing few, if any, symptoms (7). Eighty to eighty-five percent are phaeochromocytomas arising from the adrenal medulla. Fifteen to twenty percent are sympathetic paragangliomas arising from the sympathetic ganglia in the thorax, abdomen, and pelvis (8). Liang-Liang Hu et al. describe the unusual localization of a paraganglioma/pheochromocytoma to the bladder of a 52-year-old woman with paroxysmal palpitations Hu et al.

## 6. Malignant hypertension

Malignant hypertension is defined as uncontrolled severe hypertension in the presence of acute organ damage including the brain, heart, and kidney. This condition is often associated with thrombotic microangiopathy, which is an expression of endothelial acute damage (9). For this reason, it has to be differentiated from thrombotic thrombocytopenic purpura, which addresses the management of the case toward plasma exchange. Bosisio et al. describe the case of a patient affected by malignant hypertension. Prior history of hypertension, high mean arterial pressure, significant renal impairment but relatively modest thrombocytopenia, and a lack of severe ADAMTS-13 deficiency (activity <10%) at diagnosis are clues to diagnose malignant hypertension-induced thrombotic microangiopathy Bosisio et al. This very well-described case allows the reader to focus on three major issues: (1) Malignant hypertension needs to be included in the flowchart for the diagnosis in the presence of acute syndrome in order to save time and prevent further damage; (2) fast reduction of blood pressure levels might as well favor the progression of the organ damage, given the reduced perfusion of target organs such as the heart or the kidney; (3) recidivism occurs, in particular, to those patients who do not adhere to treatment; therefore, strategies for improving adherence (including closer follow-ups) need to be put in place.
